# The Italian National Project of Astrobiology—Life in Space—Origin, Presence, Persistence of Life in Space, from Molecules to Extremophiles

**DOI:** 10.1089/ast.2020.2247

**Published:** 2020-04-30

**Authors:** Silvano Onofri, Nadia Balucani, Vincenzo Barone, Piero Benedetti, Daniela Billi, Amedeo Balbi, John Robert Brucato, Beatrice Cobucci-Ponzano, Giovanna Costanzo, Nicoletta La Rocca, Marco Moracci, Raffaele Saladino, Giovanni Vladilo

**Affiliations:** ^1^ Department of Ecological and Biological Sciences, University of Tuscia, Viterbo, Italy.; ^2^ Department of Chemistry, Biology and Biotechnology, Perugia, Italy.; ^3^ Scuola Normale Superiore di Pisa, Pisa, Italy.; ^4^ Department of Biology, University of Padova, Padova, Italy.; ^5^ “Centro Linceo Beniamino Segre”, Accademia Nazionale dei Lincei, Rome, Italy.; ^6^ Department of Biology, University of Rome Tor Vergata, Rome, Italy.; ^7^ Department of Physics, University of Rome Tor Vergata, Rome, Italy.; ^8^ INAF - Arcetri Astrophysical Observatory, Florence, Italy.; ^9^ Institute of Biosciences and BioResources, CNR, Naples, Italy.; ^10^ Institute of Molecular Biology and Pathology, CNR, Rome, Italy.; ^11^ Department of Biology, University of Naples ‘Federico II’, Naples, Italy.; ^12^ INAF—Astronomical Observatory of Trieste, Trieste, Italy.

Sunt qui scire volunt ut aedificent… et caritas est

Bernard de Clairvaux

**Figure:**
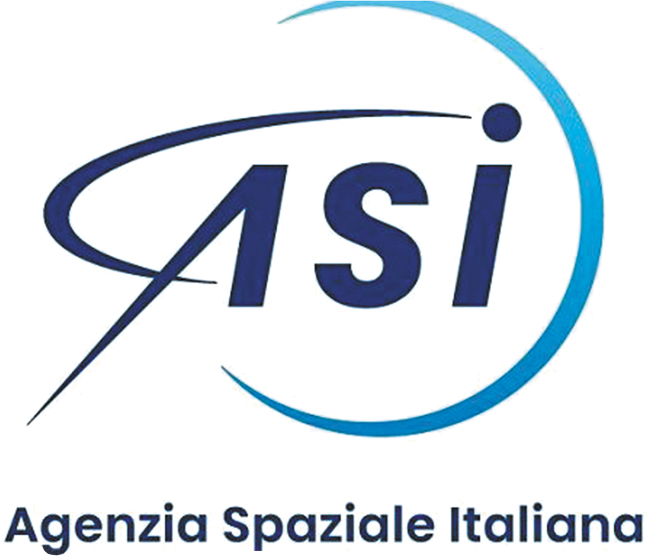


The “Life in Space” project was funded in the wake of the Italian Space Agency's proposal for the development of a network of institutions and laboratories conceived to implement Italian participation in space astrobiology experiments. Of primary concern for this project is the study of the origin of life in the Universe, a focus that will promote investigation into prebiotic chemistry in various possible scenarios, whether in polar or nonpolar solvents (*e.g.,* Titan's environment). Such results will link with study of the effects of simulated space conditions on possible chemical biosignatures. The limits of life as we know it will be investigated in ground-based experiments with microorganisms that have already demonstrated their resistance to extreme environments on Earth and to real or simulated space conditions. The potential survival of microorganisms will also be examined with up-to-date molecular methods. The ability of some microorganisms to produce atmospheric and surface biosignatures when exposed to simulated conditions will be tested and compared with the possible existence of biosignatures on potentially habitable exoplanets. Furthermore, the search for potentially habitable exoplanets, with space-based observational methods, will be optimized by way of dedicated climate models with the capacity to predict the detectability of atmospheric biosignatures for a broad range of planetary conditions.

The project embraces the four most important topics in astrobiological research, as listed below, along with relevant contributions from the participating Italian institutions.

- Origins and evolution of organic compounds of biological significance in space (comets, asteroids, rocky planets, and moons);
- Prebiotic syntheses, origin of life, and early life;
- The limits of life and biological habitability: origin, evolution and adaptation of life in extreme environments on Earth and in space;
- Biomarkers for life detection in the Solar System and on exoplanets.

**Figure:**
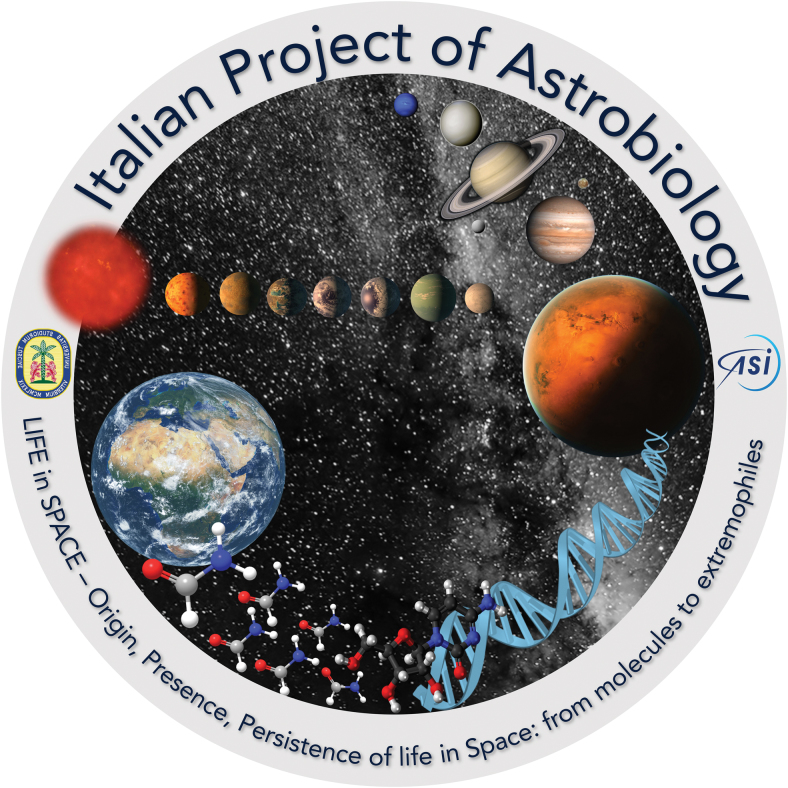


The “Origins and Evolution of Organic Compounds” topic includes the study of prebiotic organic compounds formation on ice and gas for conditions expected on Pluto, icy moons, and comets, and explores the possible synthesis of prebiotic compounds in non-aqueous solvents such as liquid methane/ethane that is present on Titan. The “Prebiotic syntheses, Origins, and Early Life” topic focuses on formamide as a precursor to more complex organic substances and will follow molecular evolution from simple chemical precursors to the basic intermediates of cellular processes, including the detection of organic life markers in planetary and space conditions. The “Limits of Life” topic will investigate the biology and limits of adaptation of extremophilic Archaea and microbiomes from extreme solfataric environments. Microorganisms and radioprotective pigments (melanin) will be tested under space-relevant radiation to study the protective effects on biological systems and materials. In addition, astrobiological models such as cyanobacteria and microfungi will be investigated by means of omics technologies under simulated Mars and icy-moon conditions and M-star simulated irradiation with the intent to elucidate the limits of life and adaptability under non-Earth conditions, as well as the permanence of biomarkers. Cyanobacteria will also be evaluated, with biochemical and omics methods, to determine the limits and mechanisms of adaptability of oxygenic photosynthetic microorganisms to simulated M-type light sources at ground conditions and increasing CO_2_ concentrations (in N_2_) up to 100% Mars-like concentrations. A data set of atmospheric biosignatures generated under a variety of tested conditions will be produced as part of the “Biomarkers for Life Detection” topic. This topic will also include laboratory irradiation studies of biomolecule interactions with mineral surfaces, identification of atmospheric and reflectance spectroscopic biomarkers of gases and pigments, respectively, from oxygenic photosynthetic extremophiles. Exoplanets that could accommodate a biosphere will also be selected for remote biosignature study.

**Figure:**
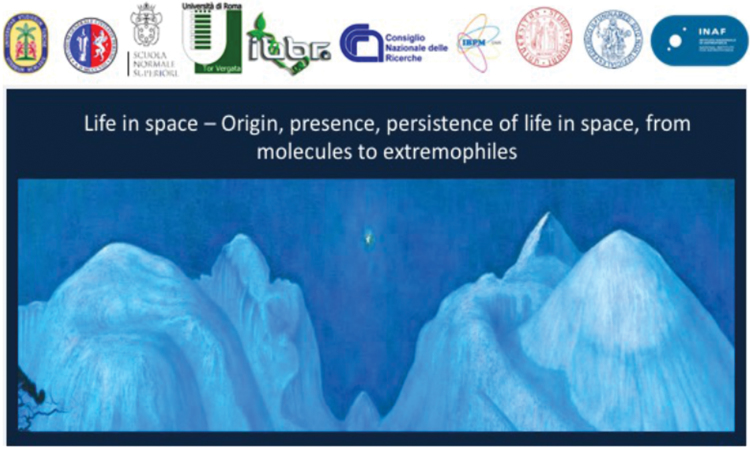


Research on these topics will provide a robust basis for possible experiments on board the International Space Station (ISS) and other space platforms, such as the lunar orbiting platform-Gateway, thus enhancing Italian competitiveness in European and international astrobiology funding opportunity announcements.

The “Life in Space” project will contribute technological innovations that have industrial application by (i) evaluating the atomistic potential and interaction of polarizable solvents via new continuum solvent models (PCM); (ii) discovering biomolecules and enzymes that are hyperstable in space; (iii) identifying efficient antioxidant mechanisms and radio-protective biomolecules; (iv) evaluating photosynthetic gases and biomass production for minimal photosynthetic light intensities; (v) utilizing oxygenic photosynthetic microorganisms for atmosphere regeneration for Bioregenerative Life Support Systems (BLSS); and (vi) testing the feasibility of utilizing a liquid micro-chromatograph with a microfluidic system for the extraction of biomolecules from planetary surfaces.

The overall effort will train at least three PhD triennial positions, support 17 annual postdoc fellowships and three researcher positions, and provide numerous projects for graduate student theses.

The impact of this Italian “Life in Space” project will be highlighted in publications and on our website http://www.lifeinspace.it.

An outreach program will include news and information published on the website, public conferences, and graphic literature for young students.

A roadmap for the future development of astrobiology in Italy will be produced upon completion of the Italian “Life in Space” program. Total funding will approach 3.5 M€ and is co-funded by the Italian Space Agency (ASI DC-VUM-2017-034).

